# Supplementary material for: NONO, SFPQ, and PSPC1 promote telomerase recruitment to the telomere
Source: Nat Commun. 2025 Jul 1;16:5769. doi: 10.1038/s41467-025-60924-w (PMC12218506; doi:10.1038/s41467-025-60924-w)
Supplement: Supplementary file 1 — Supplementary Information [file 41467_2025_60924_MOESM1_ESM.pdf]

Supplementary Tables

Supplementary Table 1. List of antibodies used in this study.

Target	Species	Source, Cat #	Immunofluorescence Dilution	Western Blot Dilution	ChIP Dilution	Immunoprecipitation Dilution
NONO	Rabbit	Sigma-Aldrich, N8789	-	1.1000	1.100	1.1000
PSPC1	Rabbit	Sigma-Aldrich, HPA038904	-	1.1000	1.50	1.1000
SFPQ	Rabbit	Abcam, ab177149	-	1.1000	-	-
SFPQ	Rabbit	Abcam, ab38148	-	1.1000	1.100	1.1000
pRPA2(S33)	Rabbit	Bethyl Laboratories, A300-246A	1.500	-	-	-
PML	Goat	Santa Cruz Biotech, sc-9862	1.500	-	-	-
TRF2	Rabbit	NOVUS, NB110-57130	-	-	1.100	-
Coilin	Mouse	Santa Cruz, sc-55594	1.500	1.2000	-	-
Actin	Rabbit	Sigma-Aldrich, A2066	-	1.5000		-
TCAB1	Rabbit	Novus Biologicals, NB100-68252	-	1.1000	-	-
Reptin	Rabbit	Cell Signalling, 8959S	-	1.1000	-	-
Pontin	Mouse	Santa Cruz Biotech, sc-393905	-	1.1000	-	-
DKC1	Rabbit	Abcam, ab156877	-	1.1000	-	-
GAR1	Rabbit	Sigma-Aldrich, HPA059098	-	1.1000	-	-
NHP2	Rabbit	Abcam, ab204352	-	1.1000	-	-
NOP10	Rabbit	Abcam, ab134902	-	1.1000	-	-
hTERT	Rabbit	Abcam, ab32020	-	1.1000	-	-
Myc-Tag (71D10)	Rabbit	Cell Signalling, 2278	-	1.5000	-	-
TIN2	Rabbit	Invitrogen, PA5-104498	-	1.1000	-	-
TPP1	Rabbit	Bethyl Laboratories, A303-069A	-	1.1000	-	-

Supplementary figures

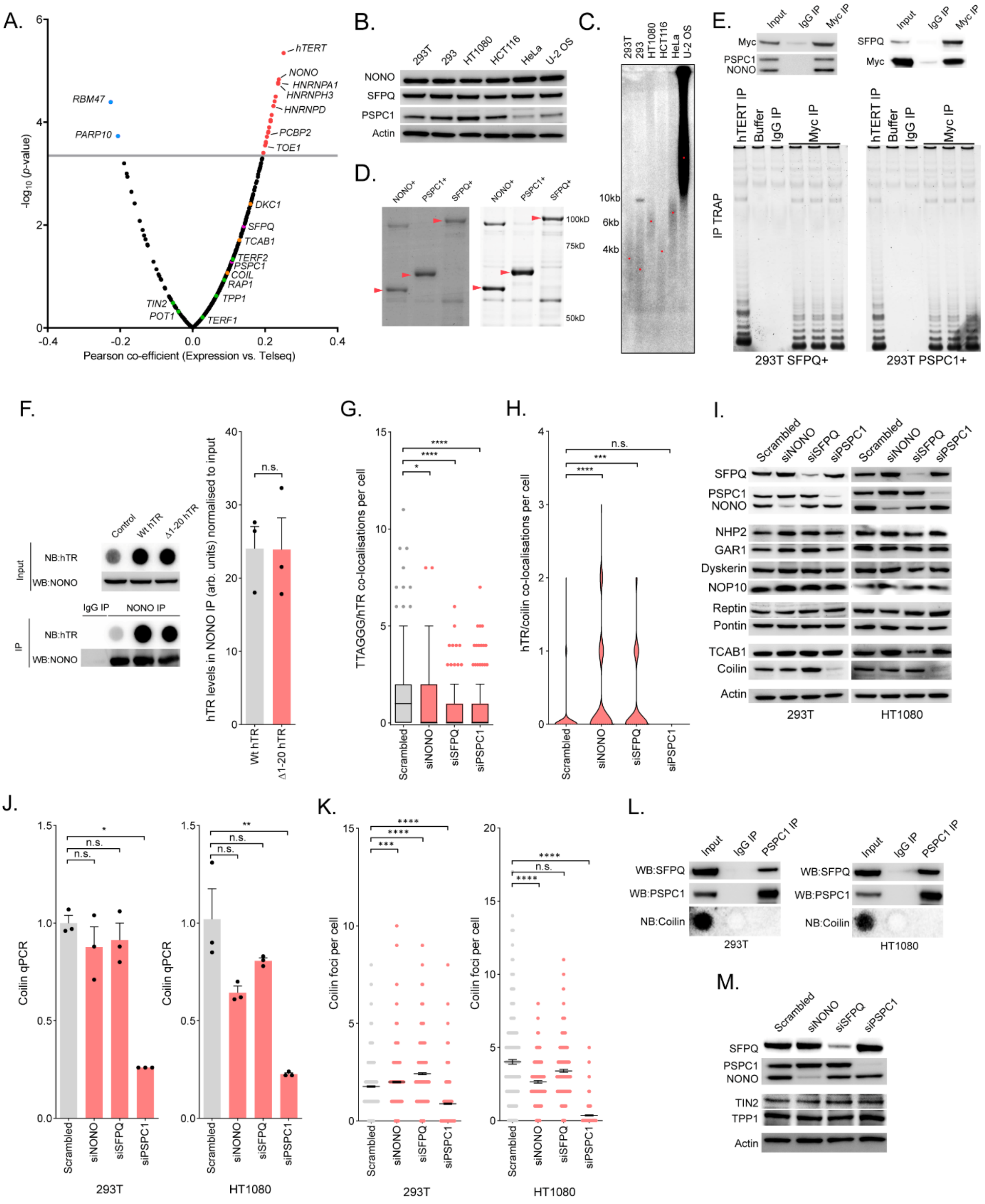

Supplementary Figure 1. RNA binding proteins associated with telomere length and the effects of DBHS protein depletion on telomerase associated protein expression. (A) Volcano plot showing Pearson co-efficient (Expression vs Telseq; x-axis) and significance (y-axis) of 402 RNA binding proteins, *COIL*, and the shelterin complex in 325 cell lines. Positive

correlations (FDR<0.01) are labelled red, negative correlations (FDR<0.01) are labelled blue, Shelterin proteins are labelled green, SFPQ and PSPC1 are labelled purple, Benjamini-Hochberg method. (B) DBHS protein levels assayed by Western blot. (C) Telomere lengths measured by TRF Southern blot (red dot indicates peak intensity). (D) Coomassie (left) and SYPRO Ruby (right) stained gels of immunopurified DBHS proteins (anti-Myc antibody) from 293T nuclear extracts. Red arrows indicate overexpressed protein. (E) TRAP on immunopurified overexpressed SFPQ and PSPC1 (anti-Myc antibody; n=3). Indicated proteins assayed by Western blot; immunopurified hTERT used as positive control. Buffer and IgG IP are negative controls. (F) Association between NONO and overexpressed hTR fragments in 293T cells (left panel). Input, NONO assayed by Western blot; wild-type (Wt) hTR and Δ1-20 hTR assayed by northern dot blot; IP, NONO associated hTR assayed by northern dot blot. IgG IP, non-specific IgG. Quantitation of hTR levels in NONO IP (right panel). Error bars represent the mean ± SEM from $n = 3$ experiments, n.s. = non-significant, Welch's t-test. (G) Tukey boxplots (median, interquartile range, and Tukey whiskers) of TTAGGG/hTR co-localizations in DBHS depleted HT1080 cells. $n = 137$ cells from three experiments, $^{*}p = 0.0119$, $^{****}p < 0.0001$, Kruskal-Wallis test. (H) Violin plot of hTR/coilin co-localizations in DBHS depleted HT1080 cells. $n = 153$ cells from three experiments, n.s. = non-significant, $^{***}p = 0.0002$, $^{****}p < 0.0001$, Kruskal-Wallis test. (I) Effects of DBHS protein depletion on telomerase associated protein expression assayed by Western blot. (J) qRT-PCR of coilin transcript levels. Values are mean ± SEM from n=3 experiments, n.s. = non-significant, $^{*}p < 0.0258$, $^{**}p = 0.0066$, Welch's t-test. (K) Tukey boxplots of Coilin foci per cell. Out of three experiments, $n = 1{,}000$ 293T and $n = 328$ HT1080 cells scored per treatment, $^{***}p = 0.0001$, $^{****}p < 0.0001$, Kruskal-Wallis test. (L) Interaction of PSPC1 with coilin transcript (PSPC1 IP). Indicated proteins assayed by Western blot; coilin transcript assayed by northern dot blot.

(M) TIN2 and TPP1 protein levels assayed by Western blot in DBHS depleted 293T cells. Source data are provided as a Source data file.

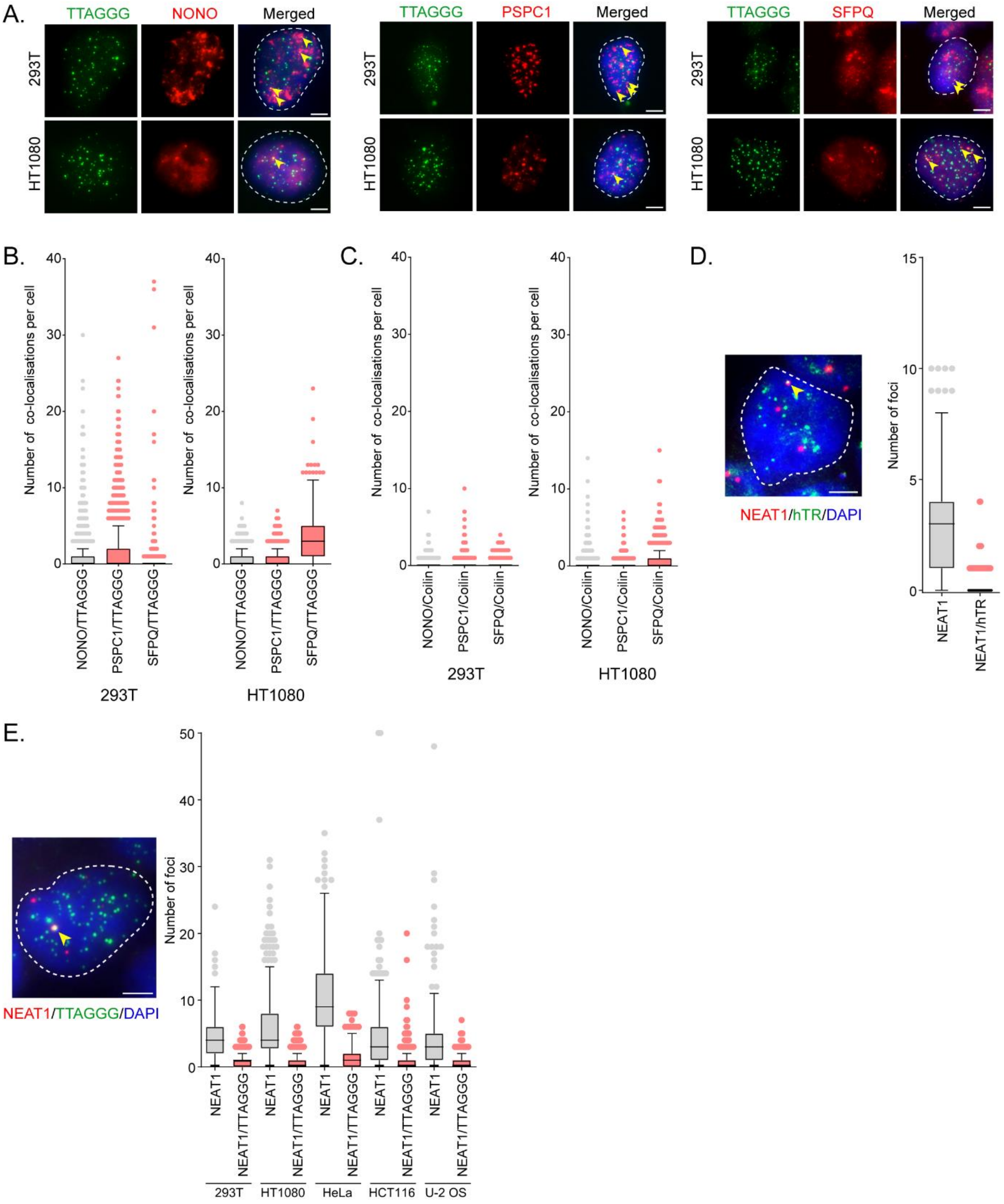

Supplementary Figure 2. Interaction of DBHS proteins and paraspeckles with telomeres, Cajal bodies, and hTR. (A) Representative images of telomere (green), and DBHS protein (red) co-localizations in 293T and HT1080 cells. DBHS/TTAGGG co-localizations are indicated by yellow arrows. Scale bars are 5 µm. (B) Tukey boxplots (median, interquartile range, and Tukey whiskers) of NONO/TTAGGG, PSPC1/TTAGGG, and SFPQ/TTAGGG co-

localizations in 293T and HT1080 cells. Out of three experiments, $n = 1{,}500$ cells in 293T and $n = 600$ cells in HT1080 were scored per treatment. (C) Tukey boxplots (median, interquartile range, and Tukey whiskers) of NONO/Coilin, PSPC1/ Coilin, and SFPQ/ Coilin co-localizations in 293T and HT1080 cells. Out of three experiments, $n = 1{,}500$ cells in 293T and $n = 600$ cells in HT1080 were scored per treatment. (D) Representative images of NEAT1 (red) and hTR (green) co-localizations in 293T cells (left panel). NEAT1/hTR co-localizations are indicated by yellow arrows. Scale bars are 5 μm. Tukey boxplots (median, interquartile range, and Tukey whiskers) of NEAT1 foci and NEAT1/hTR co-localizations (right panel). Out of three experiments, $n = 500$ cells were scored. (E) Representative images of NEAT1 (red) and telomere (green) co-localizations in 293T cells (left panel). NEAT1/TTAGGG co-localizations are indicated by yellow arrows. Scale bars are 5 μm. Tukey boxplots (median, interquartile range, and Tukey whiskers) of NEAT1 foci and NEAT1/ TTAGGG co-localizations (right panel). Out of three experiments, $n = 458$ cells were scored. Source data are provided as a Source data file.

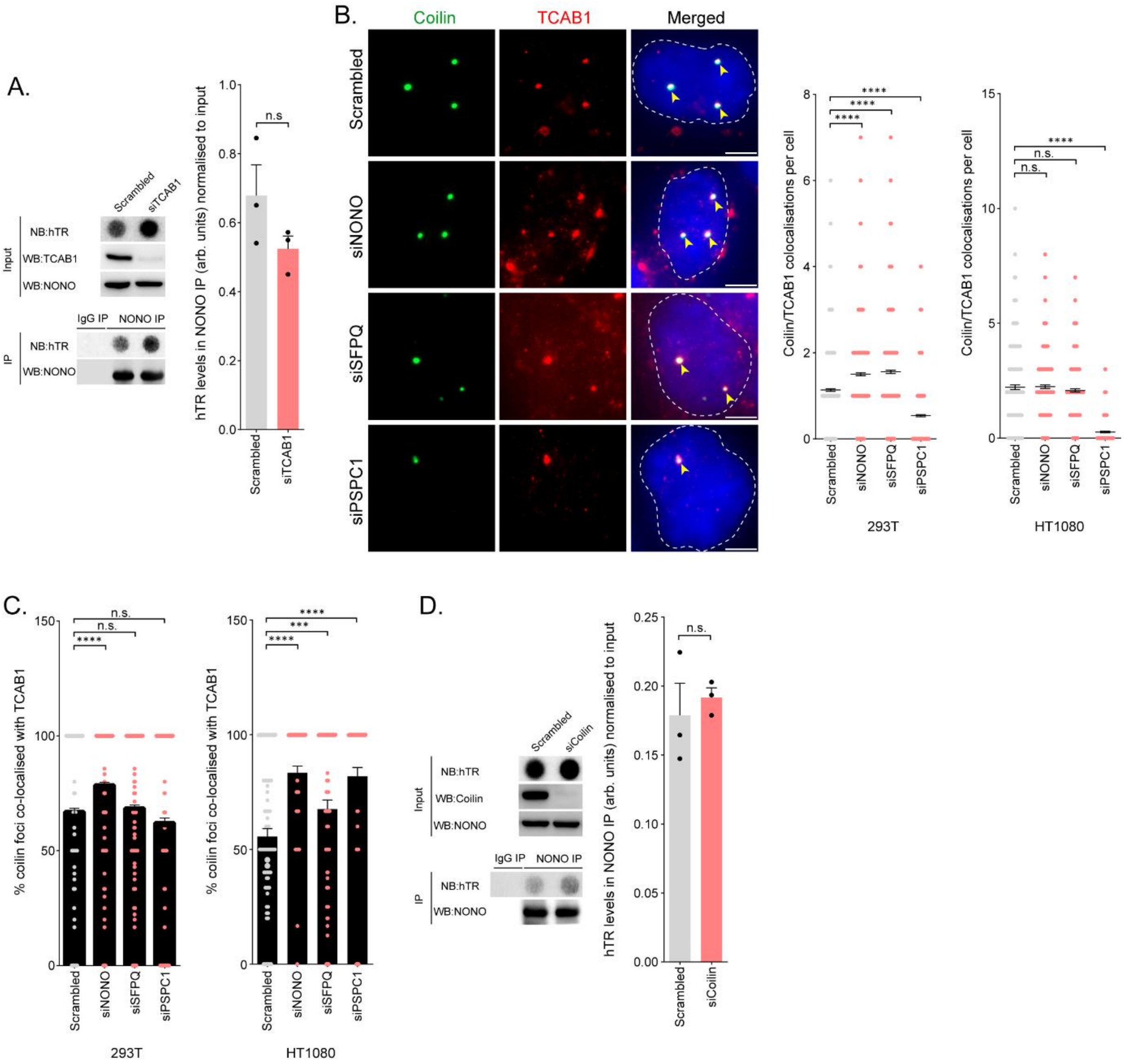

Supplementary Figure 3. Effects of DBHS protein depletion on TCAB1 and Cajal bodies. (A) Interaction of NONO with endogenous hTR assayed by IP in 293T cells depleted of TCAB1 (left panel). Input, NONO assayed by Western blot, hTR assayed by northern dot blot; IP, hTR immunopurified using NONO antibody assayed by northern dot blot. IgG IP, non-specific IgG used for IP. Quantitation of hTR levels in NONO IP (right panel). Error bars represent the mean ± SEM from $n = 3$ experiments, n.s. = non-significant, Welch's *t*-test. (B) Representative images of coilin (green) and TCAB1 (red) co-localizations in DBHS depleted 293T cells (left panel). Coilin/TCAB1 co-localizations are indicated by yellow arrows. Scale bars are 5 μm. Tukey boxplots of Coilin/TCAB1 co-localizations in 293T (middle panel) and

HT1080 (right panel). Out of three experiments, $n = 1{,}000$ 293T and $n = 327$ HT1080 cells scored per treatment, n.s. = non-significant, ****$p < 0.0001$, Kruskal-Wallis test. (C) Percentage of coilin foci co-localized with TCAB1 in 293T and HT1080 cells. Out of three experiments, $n = 516$ 293T and $n = 75$ HT1080 cells scored per treatment, n.s. = non-significant, ***$p = 0.0003$, ****$p < 0.0001$, Kruskal-Wallis test. (D) Interaction of NONO with endogenous hTR assayed by IP in 293T cells depleted of coilin (left panel). Input, NONO assayed by Western blot, hTR assayed by northern dot blot; IP, hTR immunopurified using NONO antibody assayed by northern dot blot. IgG IP, non-specific IgG used for IP. Quantitation of hTR levels in NONO IP (right panel). Error bars represent the mean ± SEM from $n = 3$ experiments, n.s. = non-significant, Welch's *t*-test. Source data are provided as a Source data file.

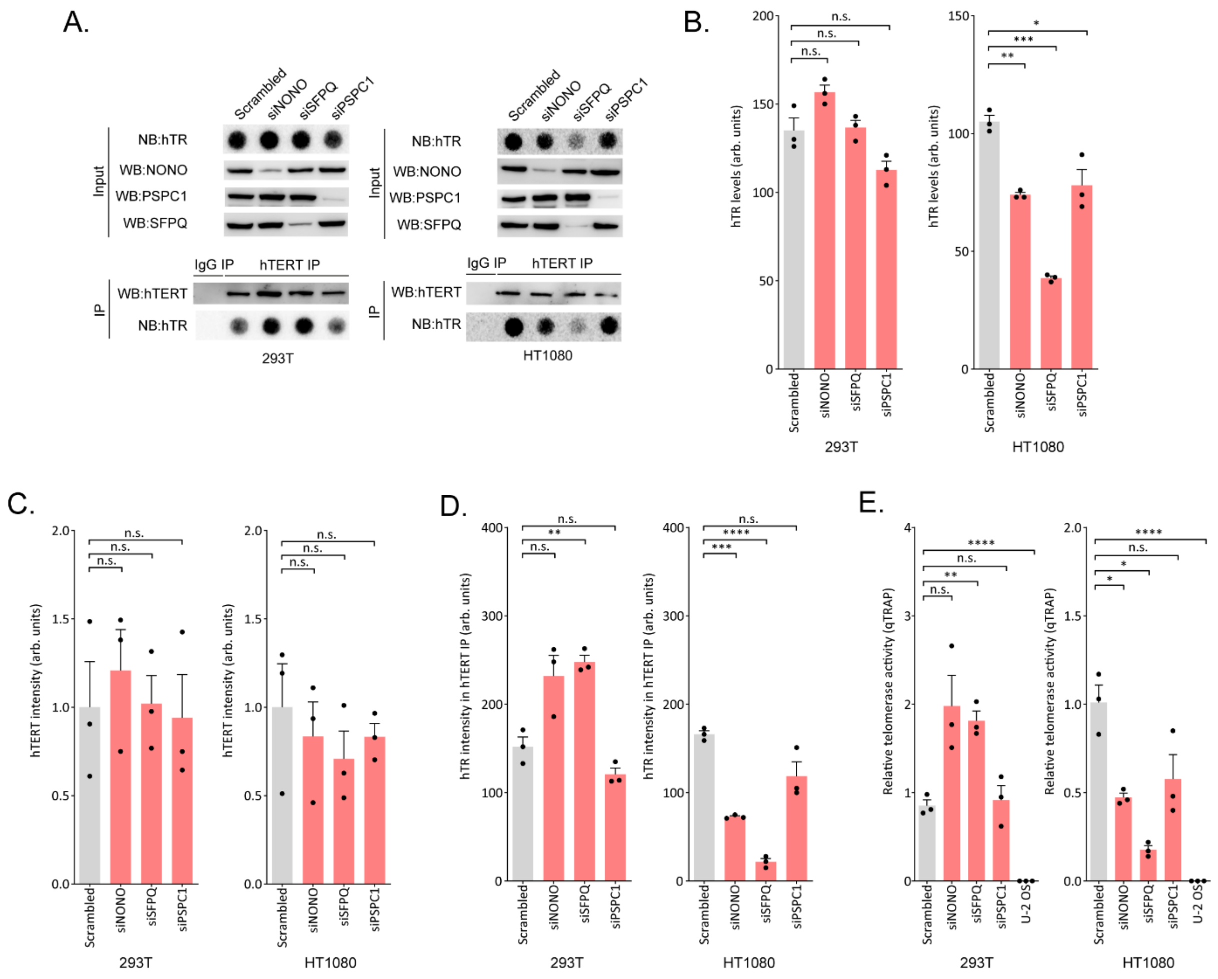

Supplementary Figure 4. Cell line specific effects of DBHS protein loss on hTR levels and telomerase activity. (A) Assembly of the telomerase catalytic core assayed by IP of hTERT and northern dot blot for hTR in DBHS protein depleted 293T and HT1080 cells. Input, indicated proteins assayed by Western blot; hTR assayed by northern dot blot. IP, telomerase RNPs immunopurified using TERT antibodies were assayed by Western blot for TERT protein and by northern dot blot for hTR. (B) Quantification of input hTR northern dot blots in DBHS depleted 293T and HT1080 cells. Values are mean ± SEM from n=3 experiments, n.s. = non-significant, $*p = 0.0413$, $**p = 0.0016$, $***p = 0.0006$ Welch's *t*-test. (C) Quantification of IP hTERT Western blots in DBHS depleted 293T and HT1080 cells. Values are mean ± SEM from n=3 experiments, n.s. = non-significant, Welch's *t*-test. (D) Assessment of assembled telomerase catalytic core complex measured through quantification of hTR levels in hTERT IP samples in DBHS depleted 293T and HT1080 cells compared to scrambled control. Values are

mean ± SEM from n=3 experiments, n.s. = non-significant, **$p = 0.0031$, ***$p = 0.0009$, ****$p < 0.0001$, Welch's *t*-test. (E) Relative telomerase activity in DBHS depleted 293T and HT1080 cells measured via qTRAP. Values are mean ± SEM from n=3 experiments, n.s. = non-significant, *$p = 0.0266$ for HT1080 Scrambled and siNONO, $p = 0.0105$ for HT1080 Scrambled and siSFPQ , **$p = 0.0038$, ****$p < 0.0001$, Welch's *t*-test. Source data are provided as a Source data file.

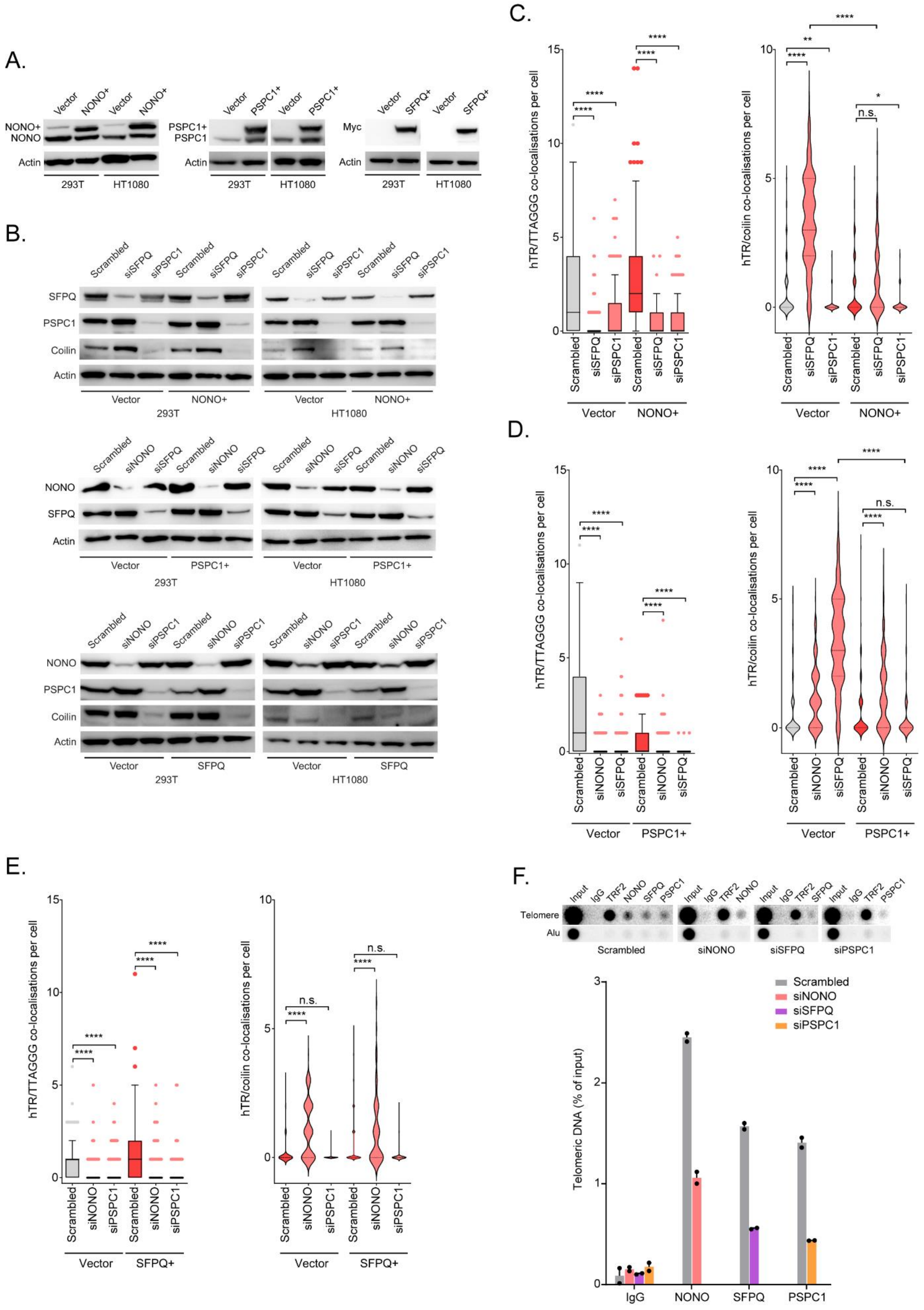

Supplementary Figure 5. Effects NONO+, SFPQ+, or PSPC1+ on telomerase recruitment in

DBHS protein depleted HT1080 cells. (A) Western blots of 293T and HT1080 cells overexpressing NONO+, SFPQ+, or PSPC1+. (B) Western blots of stable DBHS protein overexpressing 293T and HT1080 cells after siDBHS depletion. (C-E) Tukey boxplots (median, interquartile range, and Tukey whiskers) of TTAGGG/hTR co-localizations in NONO+, SFPQ+, or PSPC1+ DBHS protein depleted HT1080 cells (left panels). Out of three experiments, $n = 150$ cells scored per treatment, ****$p < 0.0001$, Kruskal-Wallis test. Violin plots of hTR/coilin co-localizations in NONO+, SFPQ+, or PSPC1+ DBHS protein depleted HT1080 cells (right panels). Out of three experiments, $n = 150$ cells scored per treatment, n.s. = non-significant, *$p = 0.0237$, **$p = 0.0038$, ****$p < 0.0001$, Kruskal-Wallis test. (F) Telomere-ChIP against DBHS proteins in 293T cells after DBHS protein knockdown. $n = 2$. Source data are provided as a Source data file.

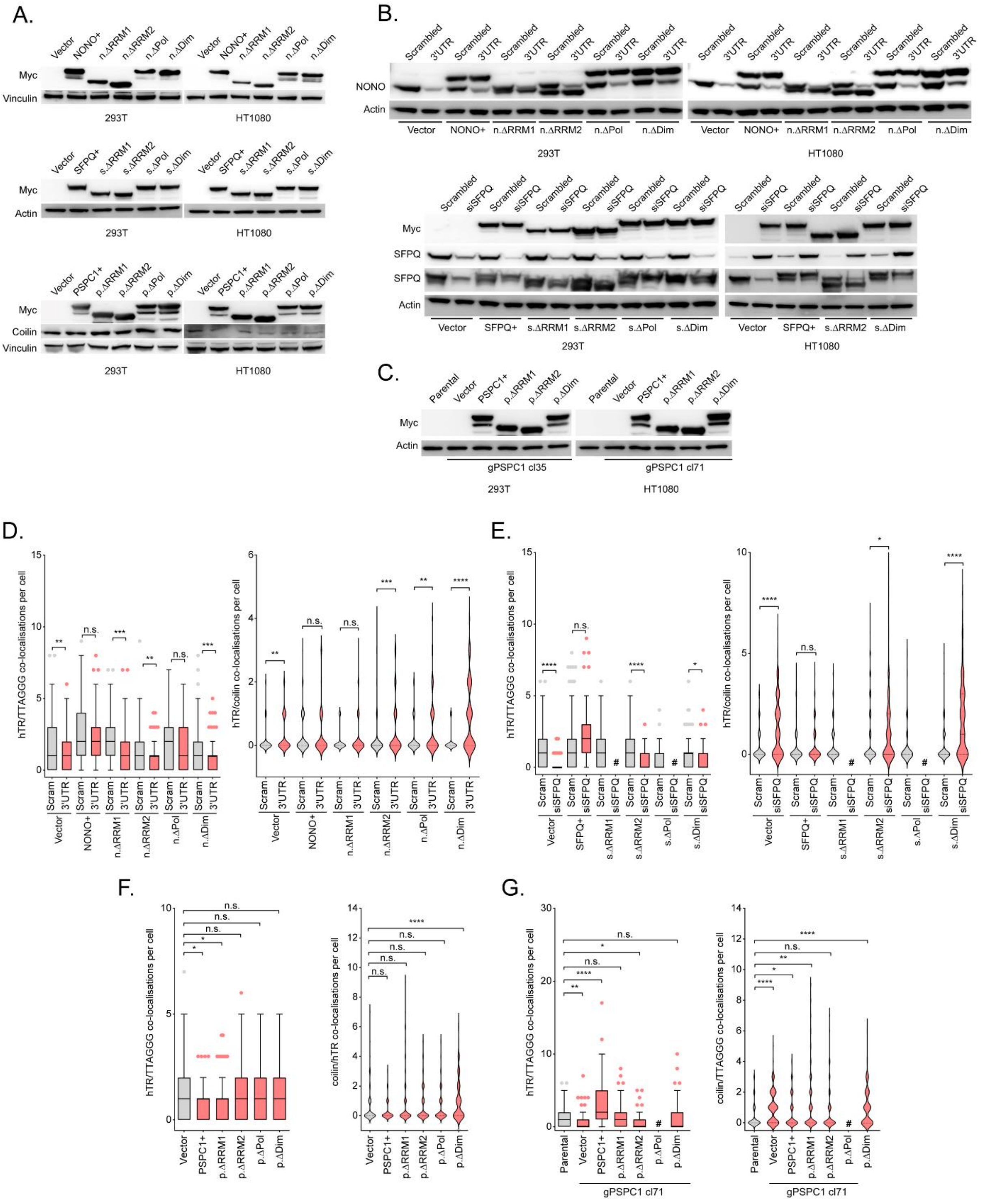

Supplementary Figure 6. DBHS protein functional mutant rescue experiments in HT1080. (A) Western blots of DBHS functional mutant stable overexpression. (B) Western blots of NONO and SFPQ functional mutant cells with siRNA depleted DBHS proteins (C) Western blots of gPSPC1 CRISPR knockout cells overexpressing PSPC1 functional mutants. (D) Tukey boxplots (median, interquartile range, and Tukey whiskers) of TTAGGG/hTR co-localizations

in HT1080 3′UTR NONO functional mutant rescue experiments (left panel). $n = 153$ cells scored per treatment from three experiments, n.s. = non-significant, $^{**}p = 0.0016$ for Vector Scram and 3′UTR, $p = 0.0039$ for n.ΔRRM2 Scram and 3′UTR, $^{***}p = 0.0010$ for n.ΔRRM1 Scram and 3′UTR, $p = 0.0002$ for n.ΔDim Scram and 3′UTR, Kruskal-Wallis test. Violin plots of hTR/coilin co-localizations in HT1080 3′UTR NONO functional mutant rescue experiments (right panel). $n = 153$ cells scored per treatment from three experiments, n.s. = non-significant, $^{**}p = 0.0092$ for Vector Scram and 3′UTR, $p = 0.0028$ for n.ΔPol Scram and 3′UTR, $^{***}p = 0.0001$, $^{****}p < 0.0001$, Kruskal-Wallis test. (E) Tukey boxplots (median, interquartile range, and Tukey whiskers) of TTAGGG/hTR co-localizations in HT1080 siSFPQ functional mutant rescue experiments (left panel). $n = 151$ cells scored per treatment from three experiments, n.s. = non-significant, $^{*}p = 0.0442$, $^{****}p < 0.0001$, Kruskal-Wallis test. Violin plots of hTR/coilin co-localizations in HT1080 siSFPQ functional mutant rescue experiments (right panel). $n = 151$ cells scored per treatment from three experiments, n.s. = non-significant, $^{*}p = 0.0133$, $^{****}p < 0.0001$, Kruskal-Wallis test. (F) Tukey boxplots (median, interquartile range, and Tukey whiskers) of TTAGGG/hTR co-localizations in HT1080 cells overexpressing PSPC1 functional mutants (left panel). $n = 150$ cells scored per treatment from three experiments, n.s. = non-significant, $^{*}p = 0.0358$ for Vector and PSPC1+, $p = 0.0494$ for Vector and p.ΔRRM1, Kruskal-Wallis test. Violin plots of hTR/coilin co-localizations in HT1080 cells overexpressing PSPC1 functional mutants (right panel). $n = 150$ cells scored per treatment from three experiments, n.s. = non-significant, $^{****}p < 0.01$, Kruskal-Wallis test. (G) Tukey boxplots (median, interquartile range, and Tukey whiskers) of TTAGGG/hTR co-localizations in HT1080 gPSPC1 cells overexpressing PSPC1 functional mutants (left panel). $n = 150$ cells scored per treatment from three experiments, n.s. = non-significant, $^{*}p = 0.0116$, $^{**}p = 0.0092$, $^{****}p < 0.0001$, Kruskal-Wallis test. Violin plots of hTR/coilin co-localizations in HT1080 gPSPC1 cells overexpressing PSPC1 functional mutants (right panel). $n = 150$ cells scored per

treatment from three experiments, n.s. = non-significant, $*p = 0.0133$, $**p = 0.0045$, $****p < 0.0001$, Kruskal-Wallis test. Source data are provided as a Source data file.

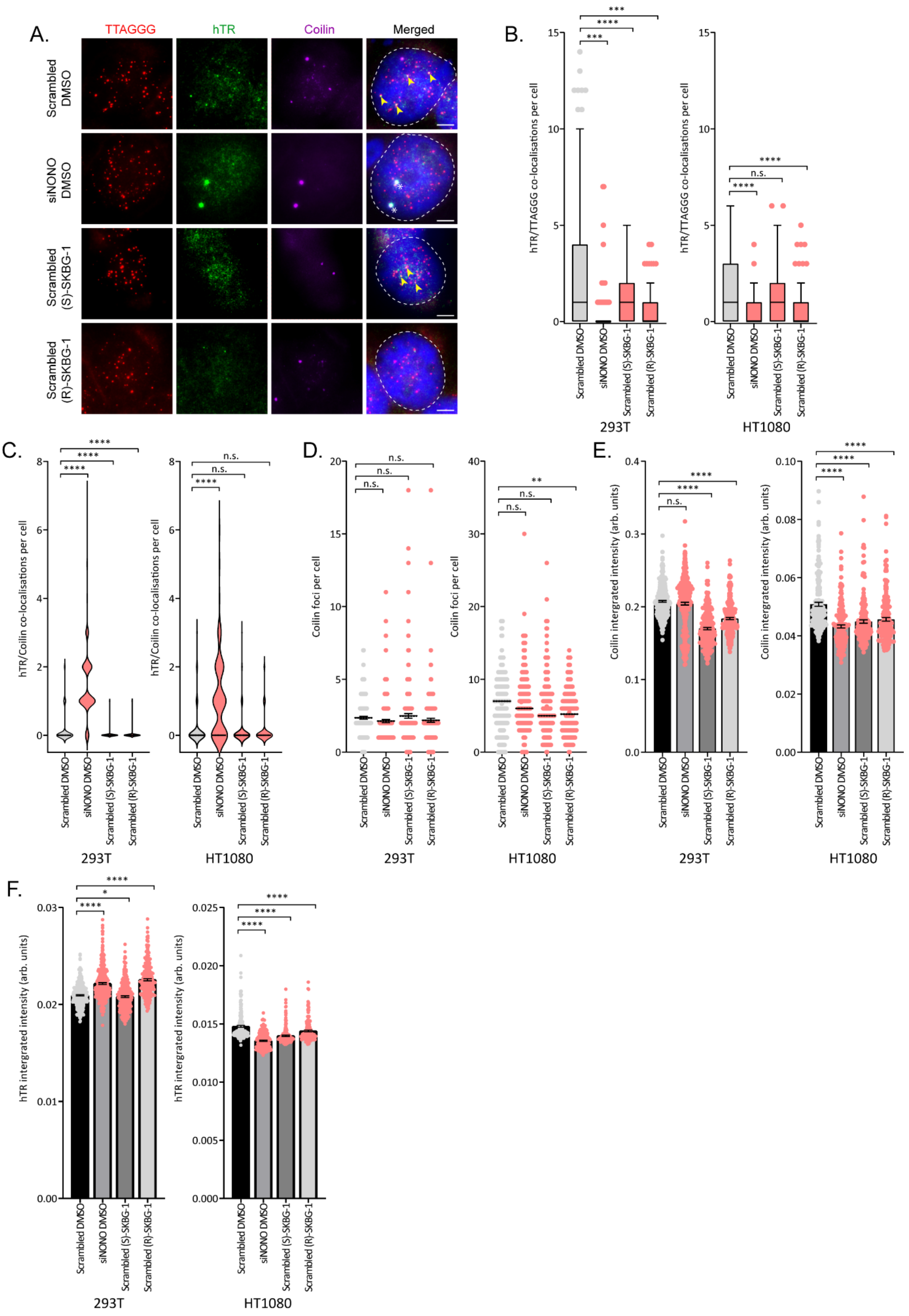

Supplementary Figure 7. Chemical inhibition of NONO using (R)-SKBG-1 impairs

telomerase recruitment to the telomere. (A) Representative images of telomere (red), hTR (green), and coilin (purple) co-localizations in 293T cells treated with 20 μM of (S)-SKBG-1 or (R)-SKBG-1 for 24 hrs. TTAGGG/hTR co-localizations are indicated by yellow arrows; hTR/coilin co-localizations are indicated by white asterisks. Scale bars are 5 μm. (B) Tukey boxplots (median, interquartile range, and Tukey whiskers) of TTAGGG/hTR co-localizations in 293T and HT1080 cells treated with 20 μM of (S)-SKBG-1 or (R)-SKBG-1 for 24 hrs. Out of three experiments, $n = 184$ 293T and $n = 103$ HT1080 cells scored per treatment, n.s. = non-significant, ***$p = 0.0007$ for 293T Scrambled DMSO and siNONO DMSO, $p = 0.003$ for Scrambled DMSO and Scrambled (R)-SKBG-1 ****$p < 0.0001$, Kruskal-Wallis test. (C) Violin plot of hTR/coilin co-localizations in 293T and HT1080 cells treated with 20 μM of (S)-SKBG-1 or (R)-SKBG-1 for 24 hrs. Out of three experiments, $n = 310$ 293T and $n = 103$ HT1080 cells scored per treatment, n.s. = non-significant, ****$p < 0.0001$, Kruskal-Wallis test. (D) Coilin foci per cell in 293T and HT1080 cells treated with 20 μM of (S)-SKBG-1 or (R)-SKBG-1 for 24 hrs. Out of three experiments, $n = 201$ 293T and $n = 167$ HT1080 cells scored per treatment, n.s. = non-significant, **$p = 0.0033$, Kruskal-Wallis test. (E) Coilin integrated intensity per cell in 293T and HT1080 cells treated with 20 μM of (S)-SKBG-1 or (R)-SKBG-1 for 24 hrs. Out of three experiments, $n = 245$ 293T and $n = 184$ HT1080 cells scored per treatment, n.s. = non-significant, ****$p < 0.0001$, Kruskal-Wallis test. (F) hTR integrated intensity per cell in 293T and HT1080 cells treated with 20 μM of (S)-SKBG-1 or (R)-SKBG-1 for 24 hrs. Out of three experiments, $n = 245$ 293T and $n = 184$ HT1080 cells scored per treatment, n.s. = non-significant, *$p < 0.0146$, ****$p < 0.0001$, Kruskal-Wallis test. Source data are provided as a Source data file.

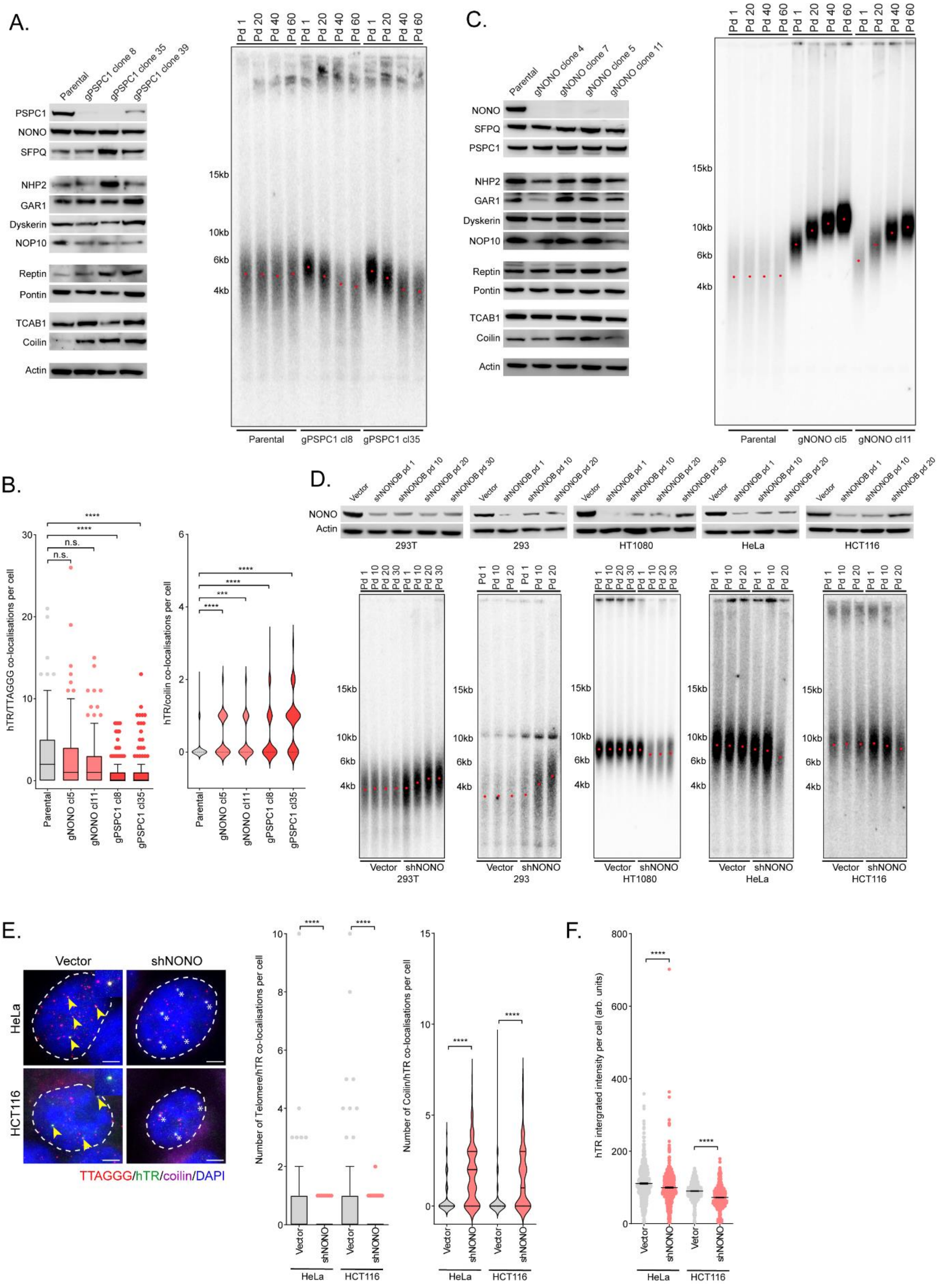

Supplementary Figure 8. Cell line specific effects of long term NONO genetic disruption. (A) Western blot of DBHS and telomerase biology associated proteins in parental 293T and

gPSPC1 CRISPR edited clones (left panel). Telomere lengths measured by TRF Southern blot with cell passage (right panel). Pd, population doublings. Peak intensity of telomere length is indicated by red dot. (B) Tukey boxplots (median, interquartile range, and Tukey whiskers) of TTAGGG/hTR co-localizations in parental 293T, gNONO, and gPSPC1 CRISPR edited clones (left panel). Out of three experiments, $n = 150$ cells scored per treatment, ****$p < 0.0001$, Kruskal-Wallis test. Violin plots of hTR/coilin co-localizations (right panel). Out of three experiments, $n = 150$ cells scored per treatment, n.s. = non-significant, ***$p = 0.0005$, ****$p < 0.0001$, Kruskal-Wallis test. (C) Western blot of DBHS and telomerase biology associated proteins in parental 293T and gNONO CRISPR edited clones (left panel). Telomere lengths measured by TRF Southern blot with cell passage (right panel). Pd, population doublings. Peak intensity of telomere length is indicated by red dot. (D) Western blot of shNONO depletion with cell passage (top panels). Telomere lengths measured by TRF Southern blot with cell passage in shNONO depleted cells (bottom panel). Pd, population doublings. Peak intensity of telomere length is indicated by red dot. (E) Representative images of telomere (red), hTR (green), and coilin (purple) co-localizations in shNONO depleted HeLa and HCT116 cells (left panel). TTAGGG/hTR co-localizations are indicated by yellow arrows; hTR/coilin co-localizations are indicated by white asterisks. Scale bars are 5 μm. Tukey boxplots (median, interquartile range, and Tukey whiskers) of TTAGGG/hTR co-localizations (middle panel). Out of three experiments, $n = 150$ cells scored per treatment, ****$p < 0.0001$, Kruskal-Wallis test. Violin plots of hTR/coilin co-localizations (right panel). Out of three experiments, $n = 150$ cells scored per treatment, ****$p < 0.0001$, Kruskal-Wallis test. (F) hTR integrated intensity per cell in shNONO depleted HeLa and HCT116 cells. Out of three experiments, $n = 695$ cells scored per treatment, n.s. = non-significant, ****$p < 0.0001$, Kruskal-Wallis test. Source data are provided as a Source data file.

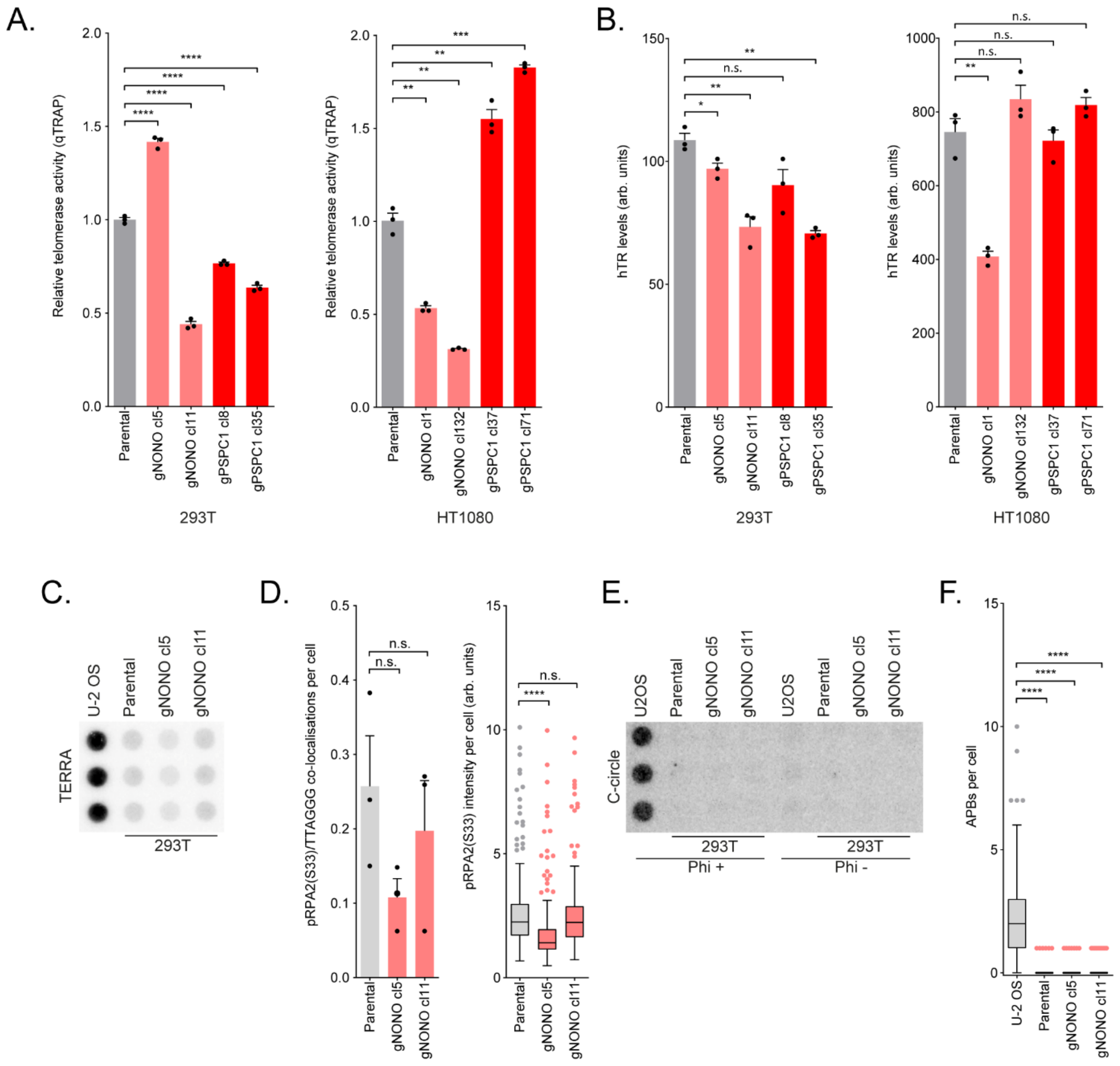

Supplementary Figure 9. Effects of NONO genetic disruption on ALT associated phenotypes in 293T cells. (A) Relative telomerase activity measured by qTRAP in gNONO and gPSPC1 CRISPR edited 293T and HT1080 clones. Values are mean ± SEM from n=3 experiments, **$p = 0.0039$ for HT1080 parental and gNONO cl1, $p = 0.0033$ for HT1080 parental and gNONO cl32, $p = 0.0014$ for HT1080 parental and gPSPC1 cl37, ****$p < 0.0001$, Welch's *t*-test. (B) Quantification of hTR northern dot blots in gNONO and gPSPC1 CRISPR edited 293T and HT1080 clones. Values are mean ± SEM from n=3 experiments, n.s. = non-significant, *$p = 0.0322$, **$p = 0.0036$ for 293T Parental and gNONO cl11, $p = 0.0016$ for 293T Parental

and gPSPC1 cl35, $p = 0.0056$ for HT1080 Parental and gNONO cl1, Welch's *t*-test. (C) Northern dot blot of TERRA levels in U-2 OS, 293T parental and gNONO CRISPR edited clones. (D) Average number of pRPA-TIF in parental 293T and gNONO CRISPR edited clones (left panel). Values are mean ± SEM from n=3 experiments, n.s. = non-significant, Welch's *t*-test. Tukey boxplots (median, interquartile range, and Tukey whiskers) of pRPA2(S33) intensity in parental 293T and gNONO CRISPR edited clones (right panel). Out of three experiments, $n = 240$ cells scored per treatment, n.s. = non-significant, ****$p < 0.0001$, Kruskal-Wallis test. (E) C-circle assay dot blots in U-2 OS, 293T parental, and gNONO CRISPR edited clones. Phi+, Phi(Φ)29 DNA polymerase added. Phi-, no Phi(Φ)29 DNA polymerase added. (F) Tukey box plots (median, interquartile range, and Tukey whiskers) of APB frequency in U-2 OS, 293T parental and gNONO CRISPR edited cells Out of three experiments, $n = 150$ cells scored per treatment, ****$p < 0.0001$, Kruskal-Wallis test. Source data are provided as a Source data file.

Supplementary Methods

Chemical synthesis

1.1 General procedures.

All reactions were conducted using dry glassware and under an inert nitrogen atmosphere unless otherwise specified. Chemical reagents were purchased from Sigma-Aldrich unless otherwise specified, and used without further purification. Anhydrous dichloromethane was obtained from a PureSolv MD7 (Inert Corporation) and dried over 3 Å molecular sieves for at least 1 h. Thin-layer chromatography was performed on aluminum-backed Silica Gel 60 F254 200 µm plates (ChemSupply). Column chromatography was performed on a Biotage® Selekt using Silica Gel 60 LR 0.04-0.06 mm (230-400 mesh ASTM, ChemSupply).

Nuclear magnetic resonance (NMR) was performed using a Bruker 500 or 600 MHz spectrometer locking to $CDCl_3$, and spectra were referenced to internal standard tetramethylsilane (δ_H 0.00 ppm) for ^1H NMR and $CDCl_3$ (δ_C 77.0 ppm) for ^{13}C NMR. Chemical shift values are reported in parts per million, ^1H-^1H coupling constants are reported in hertz and H multiplicity is abbreviated as: s = singlet, d = doublet, t = triplet, m = multiplet.

Liquid chromatography-mass spectrometry (LCMS) was performed using a Shimadzu Nexera-I LC-2040C Plus coupled to a Shimadzu LCMS-2020 mass spectrometer ESI single quadrupole mass detector (Shimadzu Corporation). Separation was performed on a Shimadzu ShimPack Velox SP-C18 column (2.1 mm × 50 mm, 2.7 µm). The mobile phases were water (solvent A) and acetonitrile (solvent B) both containing 0.1% formic acid. The gradient was 5% solvent B for 1 min, 5% to 99% solvent B over 3 min, and 99% solvent B for 2.5 min. All *m/z* values of eluting ions were measured with an ESI ion source in positive and negative ion mode and scanned between *m/z* 100-1000. Compounds were directly dissolved from solids in a 1:1 mixture of acetonitrile/water, filtered (PTFE syringe filter, 0.4 µM), then analysed by LCMS.

Specific rotation was measured in CH_2Cl_2 on an AUTOPOL III Automatic Polarimeter (Rudolph Research Analytical) using a 100 mm cell and reported with solution concentration (g/100 mL). Identical synthetic routes were used for the *R* and *S* enantiomers, using the corresponding pure enantiomers of 1-(*tert*-butyl) 3-methyl piperazine-1,3-dicarboxylate (**1a** and **1b**) as starting materials (Ambeed, Inc.).

Synthesis of enantiopure SKBG-1. The synthetic route to access the two enantiomers of SKBG-1 was based on a modification to the route published by Kathman *et al.* to avoid the reported chiral separation steps (Supplementary Figure 10)[1].

All reported yields are estimations based on crude material, as only the final compounds were subjected to full purification.

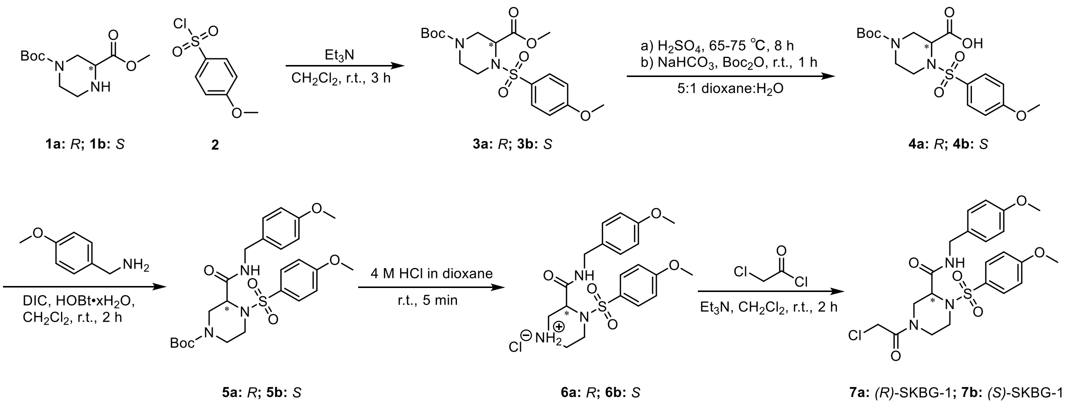

Supplementary Figure 10. Synthetic route for the enantiomers of SKBG-1, i.e. 4-(2-chloroacetyl)-*N*-(4-methoxybenzyl)-1-((4-methoxyphenyl)sulfonyl)piperazine-2-carboxamide.

Synthesis of 1-(*tert*-butyl) 3-methyl (*R*)-4-((4-methoxyphenyl)sulfonyl)piperazine-1,3-dicarboxylate (3a). 1-(*tert*-Butyl) 3-methyl (*R*)-piperazine-1,3-dicarboxylate (**1a**, 500 mg, 2.05 mmol, 1.0 equiv.) and 4-methoxybenzenesulfonyl chloride (**2**, 465 mg, 2.25 mmol, 1.1 equiv.) were dissolved in CH_2Cl_2 (4.0 mL) and triethylamine (0.14 mL, 1.0 mmol, 0.5 equiv.)

was added. After stirring for 3 h, the reaction mixture was diluted with CH_2Cl_2 (20 mL), washed with water (2 × 30 mL), brine (30 mL), dried over Na_2SO_4, and concentrated in *vacuo*, yielding the crude 1-(*tert*-butyl) 3-methyl (*R*)-4-((4-methoxyphenyl)sulfonyl)piperazine-1,3-dicarboxylate (**3a**) as a white gel (670 mg, 79%). The crude material was use directly without further purification in subsequent steps.

Synthesis of (*R*)-4-(*tert*-butoxycarbonyl)-1-((4-methoxyphenyl)sulfonyl)piperazine-2-carboxylic acid (4a). The crude 1-(*tert*-butyl) 3-methyl (*R*)-4-((4-methoxyphenyl)sulfonyl)piperazine-1,3-dicarboxylate (**3a**, 500 mg, 1.21 mmol, 1.0 equiv.) was dissolved in 5:1 dioxane/water (5 mL). The solution was cooled to 0 °C, then concentrated H_2SO_4 (3.2 mL, 60 mmol, 50 equiv.) was added dropwise in three portions over 6.5 h. Between the portions of dropwise addition, the reaction mixture was heated to 65 °C while open to air. After the final dropwise addition, the reaction mixture was heated to 75 °C for 1.5 h. The reaction mixture was then cooled to room temperature and $NaHCO_3$ was added as a solid until pH 8 was reached. After filtering through Celite and rinsing with water (5.0 mL), the filtrate was collected. To the filtrate was added di-*tert*-butyl dicarbonate (0.36 mL, 1.6 mmol, 1.3 equiv.) dropwise and the reaction was stirred at room temperature for 1 h. The reaction was then diluted with water (5.0 mL) and washed with CH_2Cl_2 (15 mL). The aqueous layer was collected and acidified to pH 2 using 1 M HCl. The crude product was extracted with CH_2Cl_2 (30 mL), and the organic layer was washed with water (2 × 30 mL), brine (30 mL), dried over Na_2SO_4, and concentrated *in vacuo* to yield the crude (*R*)-4-(*tert*-butoxycarbonyl)-1-((4-methoxyphenyl)sulfonyl)piperazine-2-carboxylic acid (**4a**) as a white gel (323 mg, 67%). The crude material was use directly without further purification in subsequent steps.

Synthesis of *tert*-butyl (*R*)-3-((4-methoxybenzyl)carbamoyl)-4-((4-methoxyphenyl)sulfonyl)piperazine-1-carboxylate (5a). The crude (*R*)-4-(*tert*-butoxycarbonyl)-1-((4-methoxyphenyl)sulfonyl)piperazine-2-carboxylic acid (**4a**, 200 mg,

0.50 mmol, 1 equiv.) was dissolved in CH_2Cl_2 (3.0 mL). *N*,*N*'-Diisopropylcarbodiimide (0.08 mL, 0.5 mmol, 1 equiv.) and 1-hydroxybenzotriazole hydrate (68 mg, 0.50 mmol, 1 equiv.) were added and the mixture was stirred for 10 min. 4-Methoxybenzylamine (0.07 mL, 0.5 mmol, 1 equiv.) was then added and the reaction was stirred for a further 2 h. The reaction mixture was diluted with CH_2Cl_2 (30 mL), washed with water (2 × 50 mL), brine (30 mL), dried over Na_2SO_4 and concentrated *in vacuo*. The crude *tert*-butyl (*R*)-3-((4-methoxybenzyl)carbamoyl)-4-((4-methoxyphenyl)sulfonyl)piperazine-1-carboxylate (**5a**) was yielded as a light yellow solid (230 mg, 88%). The crude material was use directly without further purification in subsequent steps.

Synthesis of (*R*)-3-((4-methoxybenzyl)carbamoyl)-4-((4-methoxyphenyl)sulfonyl)piperazin-1-ium chloride (6a). To crude *tert*-butyl 3-((4-methoxybenzyl)carbamoyl)-4-((4-methoxyphenyl)sulfonyl)piperazine-1-carboxylate (**5a**, 150 mg, 0.29 mmol, 1.0 equiv.), 4 M HCl in dioxane (1.0 mL, 4.3 mmol, 15 equiv.) was added and the reaction was stirred for 5 min, then nitrogen was flowed over the reaction for 15 min to remove any liberated gases. The mixture was concentrated *in vacuo* to yield the crude (*R*)-3-((4-methoxybenzyl)carbamoyl)-4-((4-methoxyphenyl)sulfonyl)piperazin-1-ium chloride (**6a**) as a yellow gum (110 mg, 84%). The crude material was use directly without further purification in subsequent steps.

Synthesis of (*R*)-4-(2-chloroacetyl)-*N*-(4-methoxybenzyl)-1-((4-methoxyphenyl)sulfonyl)piperazine-2-carboxamide (7a; *(R)*-SKBG-1). The crude 3-((4-methoxybenzyl)carbamoyl)-4-((4-methoxyphenyl)sulfonyl)piperazin-1-ium chloride (**6a,** 50 mg, 0.1 mmol, 1 equiv.) was dissolved in CH_2Cl_2 (0.5 mL). Chloroacetyl chloride (0.02 mL, 0.2 mmol, 2 equiv.) was added to the solution dropwise, followed by triethylamine (0.02 mL, 0.1 mmol, 1 equiv.). The reaction was stirred for 2 h then diluted with CH_2Cl_2 (10 mL), washed with water (2 × 30 mL), brine (30 mL), dried over Na_2SO_4 and concentrated *in vacuo*. The

crude was purified *via* flash chromatography over silica gel 60 (10 g), eluting with 3-18% MeCN in CH_2Cl_2 over 8 column volumes to yield (*R*)-4-(2-chloroacetyl)-*N*-(4-methoxybenzyl)-1-((4-methoxyphenyl)sulfonyl)piperazine-2-carboxamide (**7a,** 32 mg, 54%) as a white powder.

^{1}H NMR (600 MHz, $CDCl_3$) δ 7.70 (ddd, *J* = 8.9, 3.0, 2.0 Hz, 2H), 7.05 (d, *J* = 8.6 Hz, 2H), 6.94 (ddd, *J* = 8.9, 2.9, 1.9 Hz, 2H), 6.84–6.77 (m, 3H), 4.45 (d, *J* = 12.7 Hz, 1H), 4.43–4.38 (m, 2H), 4.29 (d, *J* = 5.8 Hz, 2H), 4.20 (d, *J* = 13.6 Hz, 1H), 4.02 (d, *J* = 12.7 Hz, 1H), 3.83 (s, 3H), 3.78 (d, *J* = 14.5 Hz, 1H), 3.74 (s, 3H), 3.03 (td, *J* = 13.3, 3.5 Hz, 1H), 2.80 (dd, *J* = 14.3, 4.4 Hz, 1H), 2.37 (td, *J* = 12.0, 3.4 Hz, 1H); ^{13}C NMR (151 MHz, $CDCl_3$) δ 167.3, 166.0, 163.8, 159.2, 130.6, 129.2 (overlapping peaks), 128.9, 115.1, 114.3, 56.3, 55.8, 55.3, 44.0, 43.6, 42.9, 41.0, 40.1. The chemical shifts match the data previously reported[1].

LCMS (ESI) at retention time t_R = 3.0 min, mass calcd for $C_{22}H_{26}ClN_3O_6S$ $[M+H]^+$ 496.13, found 496.15 (error 40.31 ppm). Specific rotation $[\alpha]_D^{32}$ 10.51 (*c* 1.82, CH_2Cl_2).

Synthesis of (*S*)-4-(2-chloroacetyl)-*N*-(4-methoxybenzyl)-1-((4-methoxyphenyl)sulfonyl)piperazine-2-carboxamide (7b; *(S)*-SKBG-1). An identical synthetic procedure was followed starting from 1-(*tert*-butyl) 3-methyl (*S*)-piperazine-1,3-dicarboxylate (**1b,** 500 mg, 2.05 mmol, 1.0 equiv.), yielding the (*S*)-4-(2-chloroacetyl)-*N*-(4-methoxybenzyl)-1-((4-methoxyphenyl)sulfonyl)piperazine-2-carboxamide (**7b**, 35 mg) as a white powder.

^{1}H NMR (600 MHz, $CDCl_3$) δ 7.70 (ddd, *J* = 8.9, 3.0, 2.0 Hz, 2H), 7.05 (d, *J* = 8.6 Hz, 2H), 6.94 (ddd, *J* = 8.9, 2.9, 1.9 Hz, 2H), 6.84–6.77 (m, 3H), 4.45 (d, *J* = 12.7 Hz, 1H), 4.43–4.38 (m, 2H), 4.29 (d, *J* = 5.8 Hz, 2H), 4.20 (d, *J* = 13.6 Hz, 1H), 4.02 (d, *J* = 12.7 Hz, 1H), 3.83 (s, 3H), 3.78 (d, *J* = 14.5 Hz, 1H), 3.74 (s, 3H), 3.03 (td, *J* = 13.3, 3.5 Hz, 1H), 2.80 (dd, *J* = 14.3, 4.4 Hz, 1H), 2.37 (td, *J* = 12.0, 3.4 Hz, 1H); ^{13}C NMR (151 MHz, $CDCl_3$) δ 167.3, 166.0, 163.8,

159.2, 130.6, 129.2 (overlapping peaks), 128.9, 115.1, 114.3, 56.3, 55.8, 55.3, 44.0, 43.6, 42.9, 41.0, 40.1. The chemical shifts match the data previously reported[1].

LCMS (ESI) at retention time t_R = 3.0 min, mass calcd for $C_{22}H_{26}ClN_3O_6S$ $[M+H]^+$ 496.13, found 496.15 (error 40.31 ppm). Specific rotation $[\alpha]_D^{33}$ -9.38 (*c* 1.13, CH_2Cl_2).

1.2 Mosher analysis for validating enantiopurity. Synthesis began with the enantiomerically pure (*ee* > 95%) forms of 1-(*tert*-butyl) 3-methyl piperazine-1,3-dicarboxylate, and all synthetic steps were carried out under neutral or acidic condition (pH ≤ 7) to avoid loss of the alpha proton and subsequent the epimerisation at the stereocentre.

The enantiopurity of penultimate compounds **6a** and **6b** were determined to validate that the stereocentre had been preserved during the synthesis. The corresponding diastereotopic Mosher amides **8a** and **8b** were synthesised using the same amide coupling conditions used earlier in the synthetic route (Supplementary Figure 11), as the stereocenter was predicted to be most potentially susceptible to epimerisation under these conditions due to the electron withdrawing nature of the HOBt ester intermediate.

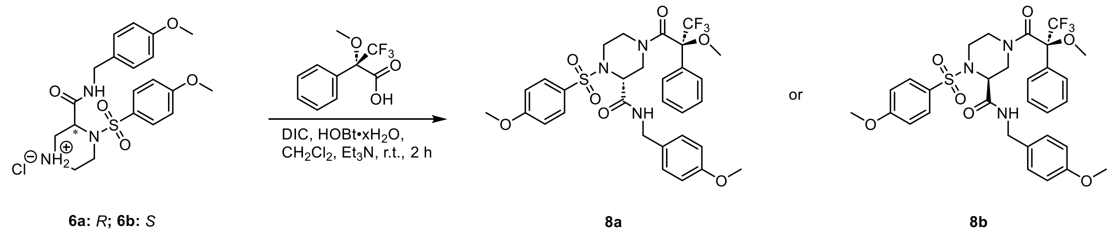

Supplementary Figure 11. Synthetic route of the Mosher amides **8a** and **8b**.

Synthesis of *(R)*-*N*-(4-methoxybenzyl)-1-((4-methoxyphenyl)sulfonyl)-4-((*S*)-3,3,3-trifluoro-2-methoxy-2-phenylpropanoyl)piperazine-2-carboxamide (8a). (*S*)-3,3,3-Trifluoro-2-methoxy-2-phenylpropanoic acid (5 mg, 0.02 mmol, 1.1 equiv.) was dissolved in CH_2Cl_2 (1 mL) and *N*,*N*'-diisopropylcarbodiimide (0.02 mL, 0.02 mmol, 1.1 equiv.) and 1-hydroxybenzotriazole hydrate (5 mg, 0.04 mmol, 1.9 equiv.) were added. After the reaction

mixture was stirred for 5 min, crude *(R)*-3-((4-methoxybenzyl)carbamoyl)-4-((4-methoxyphenyl)sulfonyl)piperazin-1-ium chloride (**6a,** 8 mg, 0.02 mmol, 1.0 equiv.) and triethylamine (0.02 mL, 0.02 mmol, 1.0 equiv.) were added. The reaction mixture was stirred for 1 h, then diluted with CH_2Cl_2 (10 mL), washed with water (2 × 20 mL), brine (20 mL), dried over Na_2SO_4 and conocentrated *in vacuo*, yielding the crude (*R*)-*N*-(4-methoxybenzyl)-1-((4-methoxyphenyl)sulfonyl)-4-((*S*)-3,3,3-trifluoro-2-methoxy-2-phenylpropanoyl)piperazine-2-carboxamide (**8a**) as a pale yellow solid (7 mg, 60%).

LCMS (ESI) at retention time t_R = 3.3 min, mass calcd for $C_{30}H_{32}F_3N_3O_7S$ $[M+H]^+$ 636.20, found 636.25 (error 78.59 ppm).

Synthesis of (*S*)-*N*-(4-methoxybenzyl)-1-((4-methoxyphenyl)sulfonyl)-4-((*S*)-3,3,3-trifluoro-2-methoxy-2-phenylpropanoyl)piperazine-2-carboxamide (8b). From the starting material **6b** (8 mg, 0.02 mmol, 1.0 equiv.), using the same synthetic procedure as for **8a** yielded the crude (*S*)-*N*-(4-methoxybenzyl)-1-((4-methoxyphenyl)sulfonyl)-4-((*S*)-3,3,3-trifluoro-2-methoxy-2-phenylpropanoyl)piperazine-2-carboxamide (**8b**) as a pale yellow solid (8 mg, 70%).

LCMS (ESI) at retention time t_R = 3.6 min, mass calcd for $C_{30}H_{32}F_3N_3O_7S$ $[M+H]^+$ 636.20, found 636.20.

NMR analysis of 8a and 8b. Crude **8a** and **8b** were each dissolved in $CDCl_3$ (0.5 mL) without further purification, to ensure that the diastereomers formed were retained for quantitative analysis. Quantitative ^{1}H NMR experiments were performed at 300 K, using D1 = 10 s and NS = 32. Both spectra were referenced to TMS at 0.00 ppm. While the spectrum of **8a** showed several impurities, the characteristic diastereotopic protons with clear integrations and splitting patterns that closely match those found in the final SKBG-1 products **7a** and **7b** (boxed) on the piperazine had distinct shifts for the two diastereomers (Supplementary Figure 12). Figures

Supplementary Figure 13 and Supplementary Figure 14 show the aliphatic regions of the NMR spectra **8a** and **8b** with chemical shifts and integration labelled, with the clear non-overlapping signals validating the enantiopurity of **6a** and **6b**.

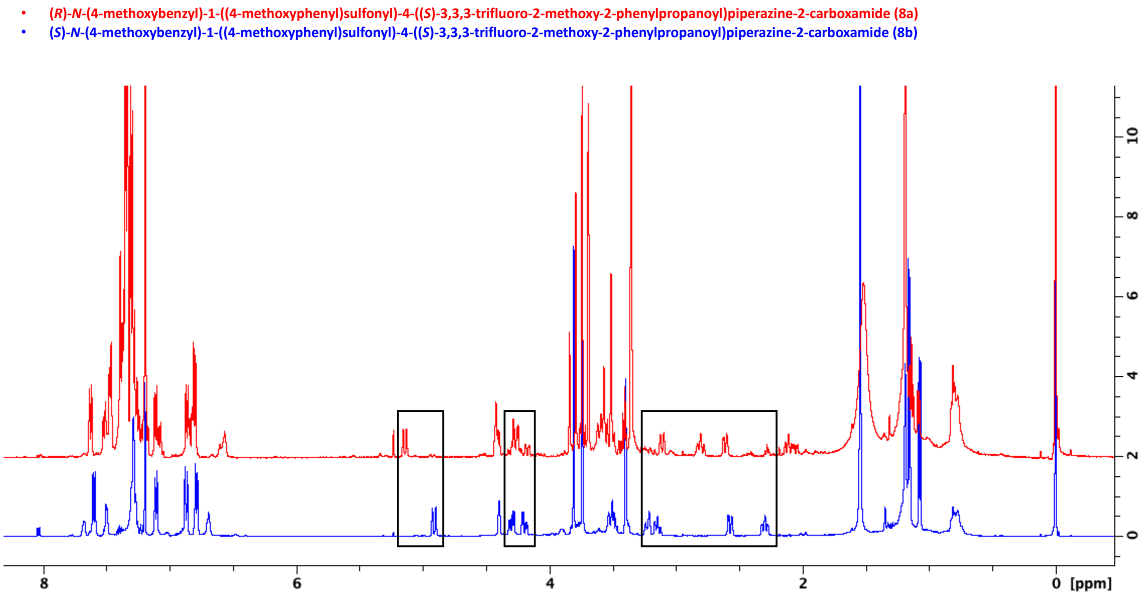

Supplementary Figure 12. Overlaid ^{1}H NMR spectra of **8a** (red) and **8b** (blue). The boxed regions contain the diastereotopic proton signals.

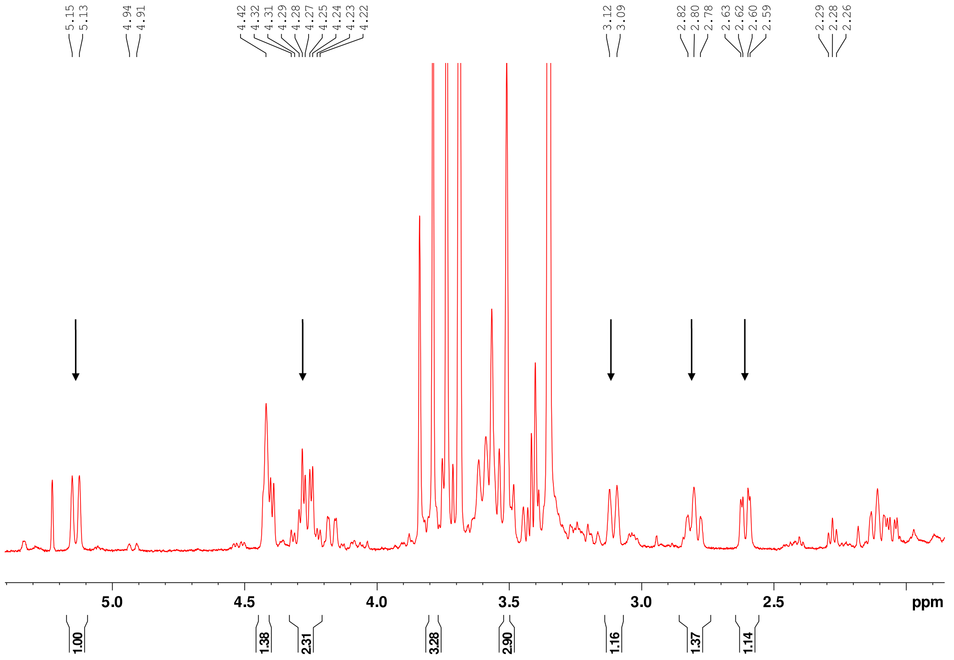

Supplementary Figure 13. A zoom-in view of the ^{1}H NMR spectrum of **8a**. Arrows indicate diagnostic signals for protons on the piperazine ring.

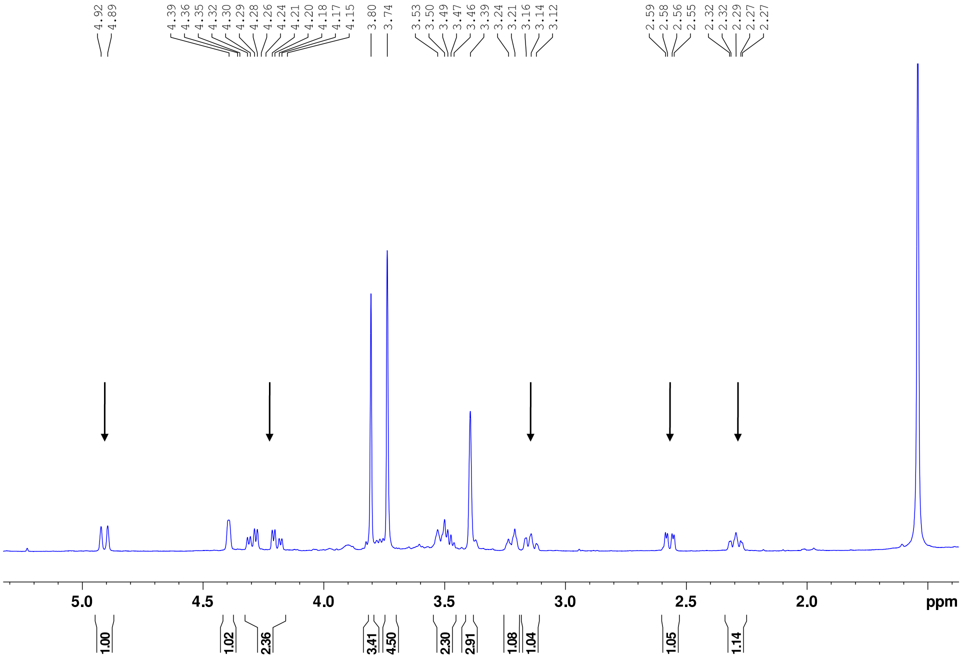

Supplementary Figure 14. A zoom-in view of the ^{1}H NMR spectrum of **8b**. Arrows indicate diagnostic signals for protons on the piperazine ring.

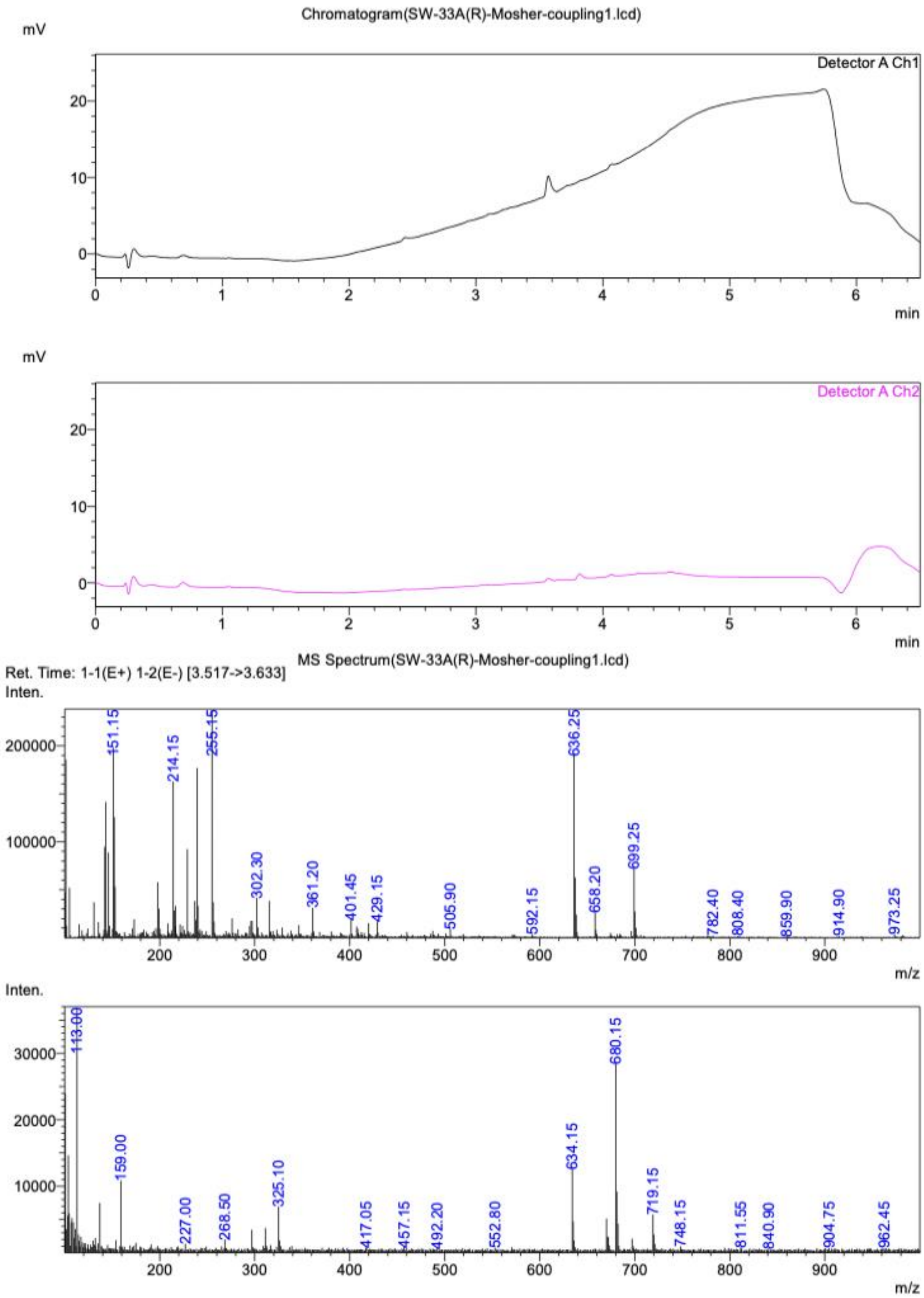

Supplementary Figure 15. LCMS analysis of **8a**, showing UV absorbance at 220 and 254 nm respectively (top), and ESI MS in positive and negative modes (bottom).

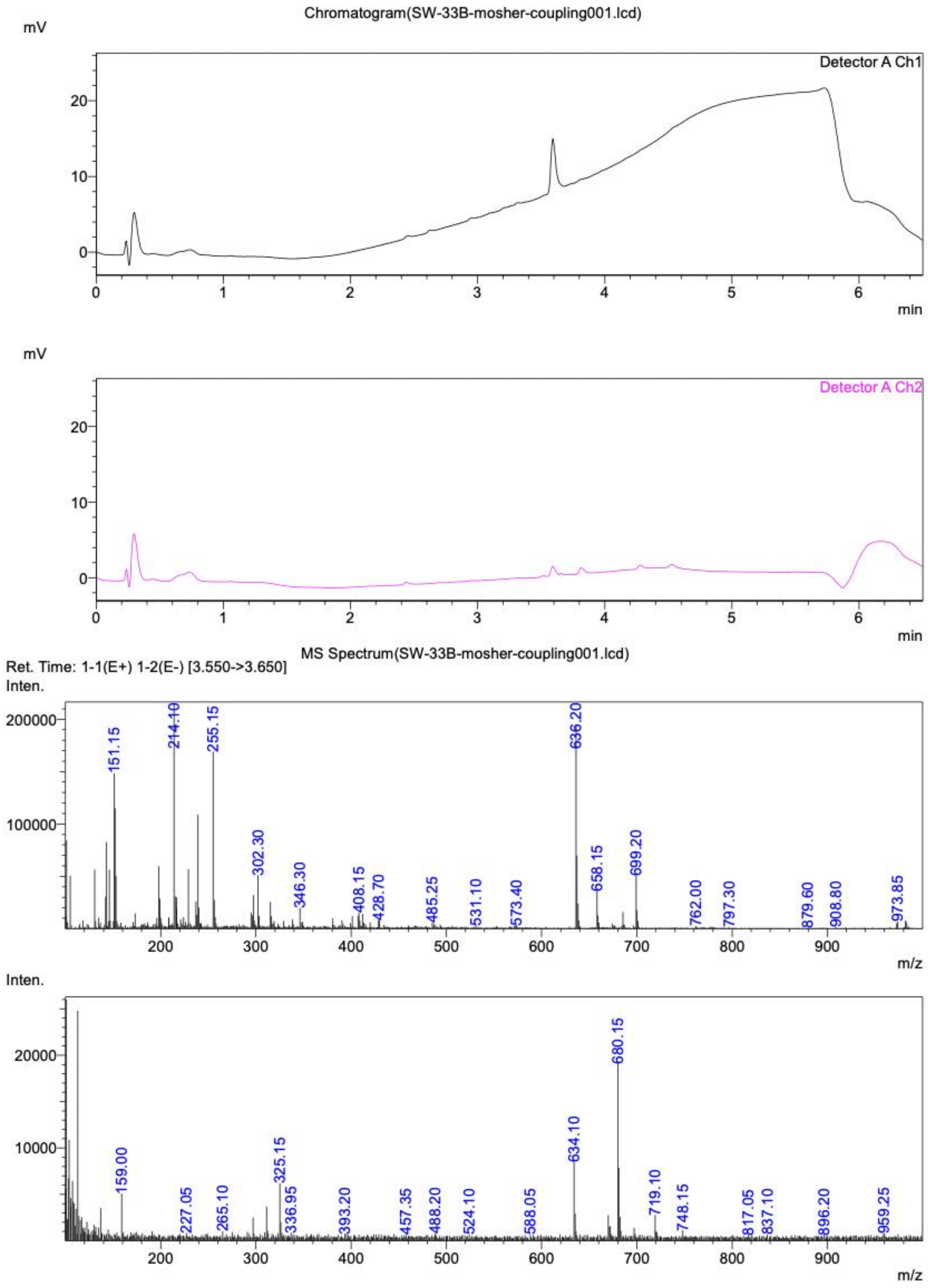

Supplementary Figure 16. LCMS analysis of **8b**, showing UV absorbance at 220 and 254 nm respectively (top), and ESI MS in positive and negative modes (bottom).

1.3 Characterisation of final compounds 7a *(R)*-SKBG-1 (Supplementary Figure 17, 18, 21) and 7b *(S)*-SKBG-1 (Supplementary Figure 19, 20, 22).

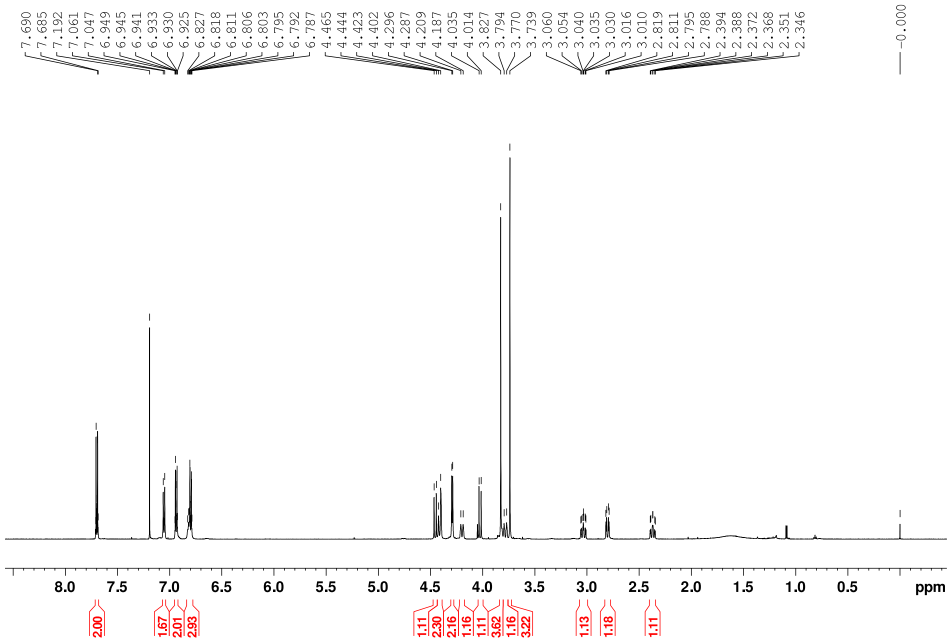

Supplementary Figure 17. ^{1}H NMR spectrum of (*R*)-4-(2-chloroacetyl)-*N*-(4-methoxybenzyl)-1-((4-methoxyphenyl)sulfonyl)piperazine-2-carboxamide (**7a**).

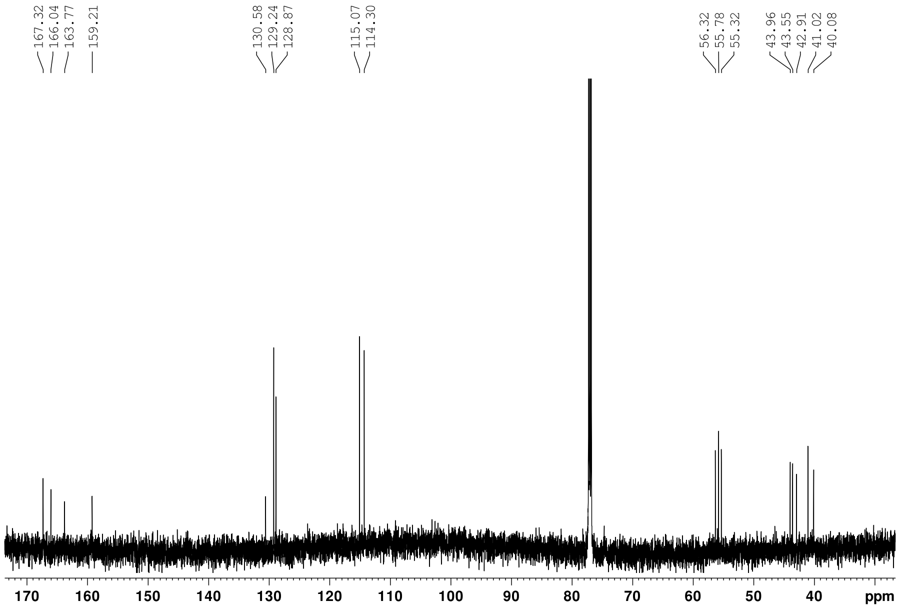

Supplementary Figure 18. ^{13}C NMR spectrum of (*R*)-4-(2-chloroacetyl)-*N*-(4-methoxybenzyl)-1-((4-methoxyphenyl)sulfonyl)piperazine-2-carboxamide (**7a**).

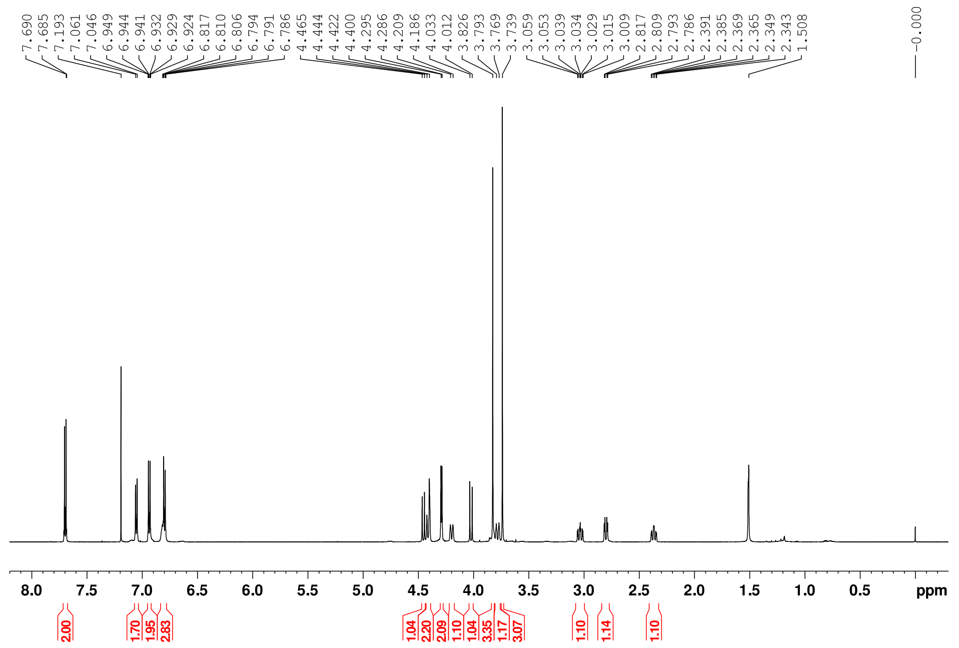

Supplementary Figure 19. ^{1}H NMR spectrum of (*S*)-4-(2-chloroacetyl)-*N*-(4-methoxybenzyl)-1-((4-methoxyphenyl)sulfonyl)piperazine-2-carboxamide (**7b**).

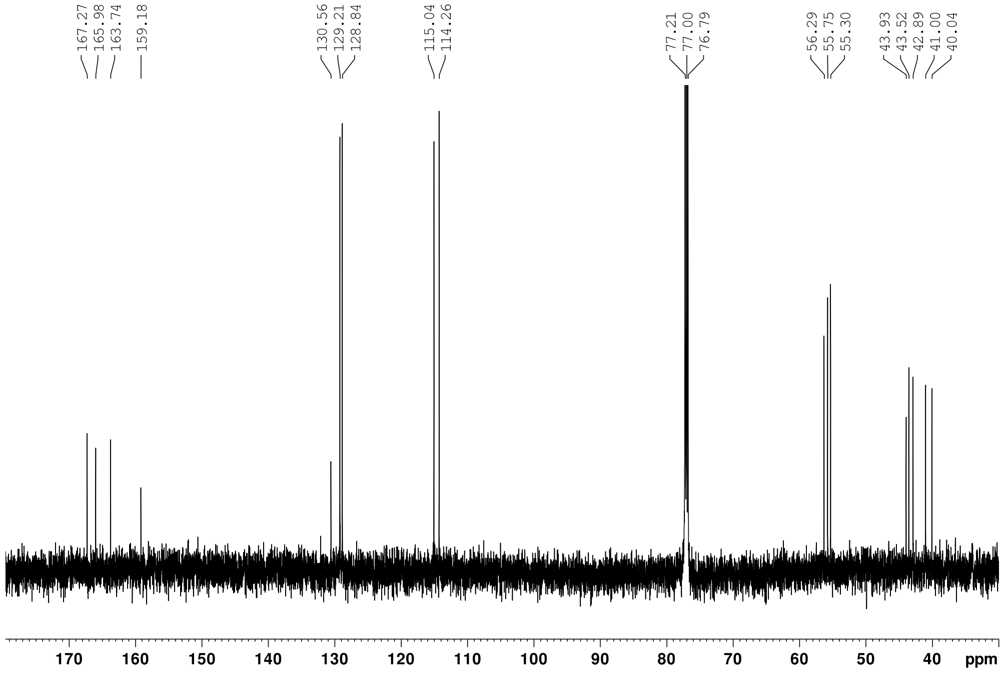

Supplementary Figure 20. ^{13}C NMR spectrum of (*S*)-4-(2-chloroacetyl)-*N*-(4-methoxybenzyl)-1-((4-methoxyphenyl)sulfonyl)piperazine-2-carboxamide (**7b**).

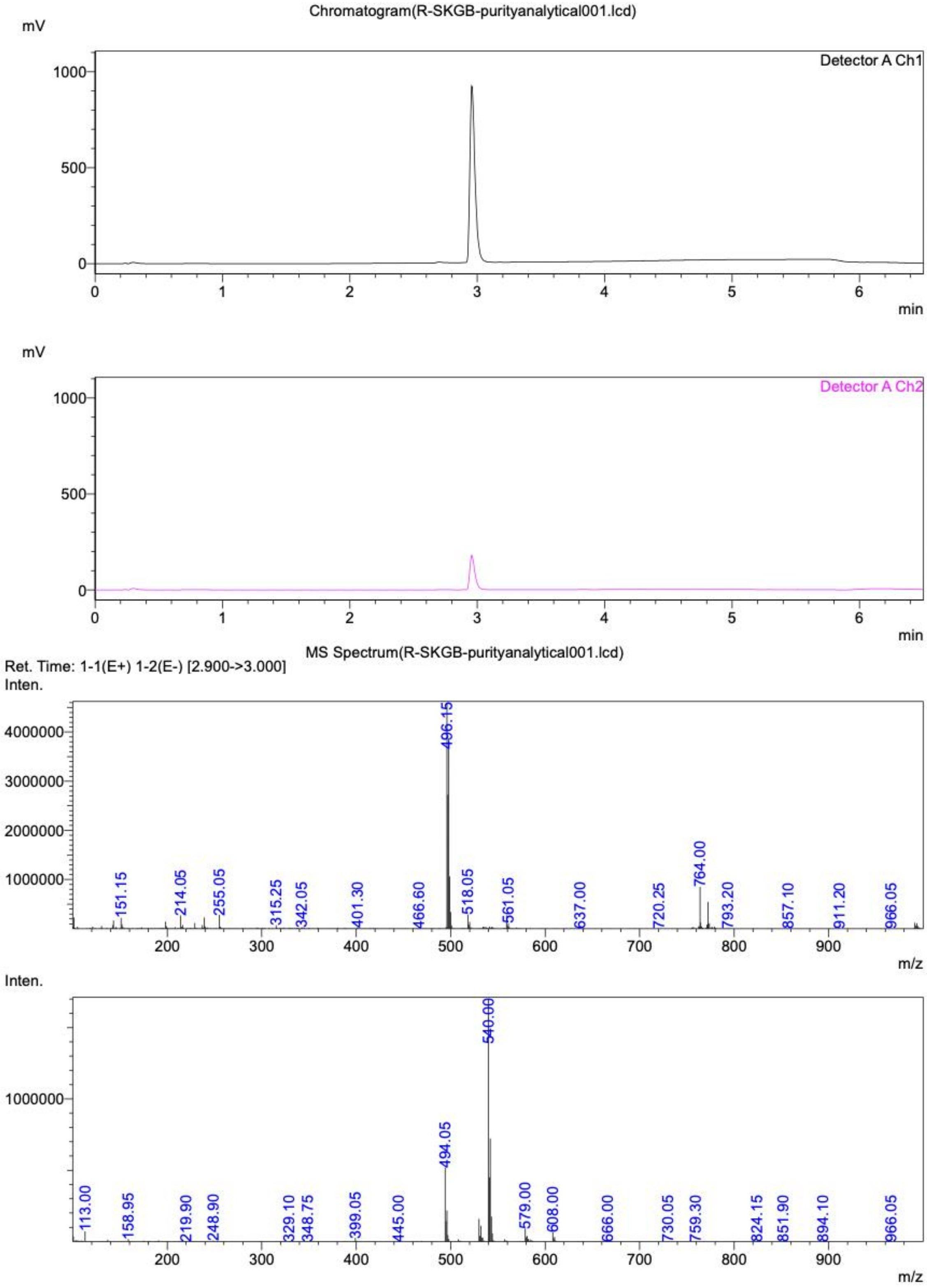

Supplementary Figure 21. LCMS analysis of **7a**, showing UV absorbance at 220 and 254 nm respectively (top), and ESI MS in positive and negative modes (bottom).

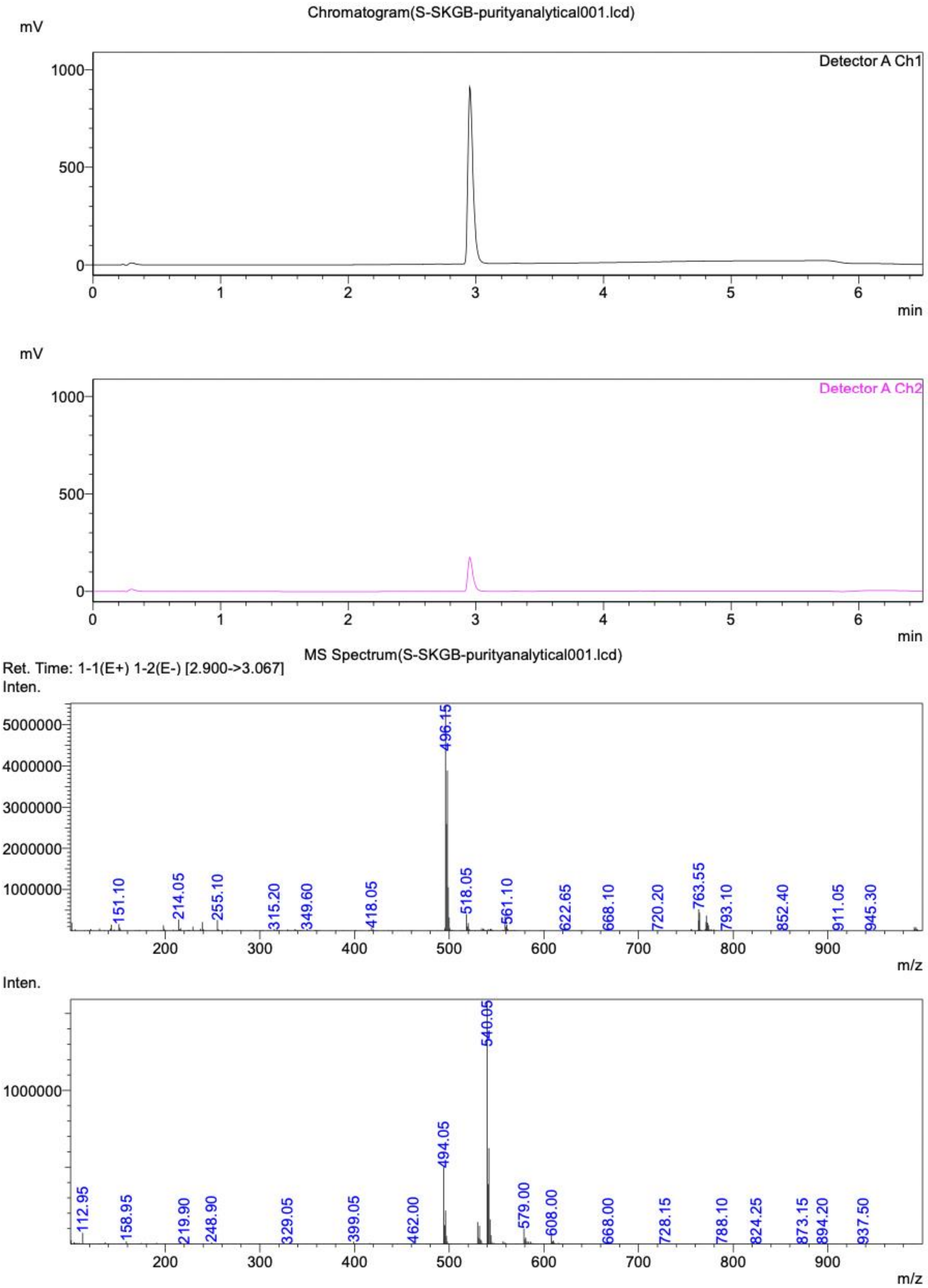

Supplementary Figure 22. LCMS analysis of **7b**, showing UV absorbance at 220 and 254 nm respectively (top), and ESI MS in positive and negative modes (bottom).

Supplementary References

1. Kathman, S.G., Koo, S.J., Lindsey, G.L., Her, H.L., Blue, S.M., Li, H., Jaensch, S., Remsberg, J.R., Ahn, K., Yeo, G.W., et al. (2023). Remodeling oncogenic transcriptomes by small molecules targeting NONO. Nat Chem Biol *19*, 825-836. 10.1038/s41589-023-01270-0.
